# Pilot Study of Remote Ischemic Conditioning in Acute Spontaneous Intracerebral Hemorrhage

**DOI:** 10.3389/fnins.2022.791035

**Published:** 2022-05-12

**Authors:** Abbas Jarrahi, Manan Shah, Meenakshi Ahluwalia, Hesam Khodadadi, Kumar Vaibhav, Askiel Bruno, Babak Baban, David C. Hess, Krishnan M. Dhandapani, John R. Vender

**Affiliations:** ^1^Department of Neurosurgery, Medical College of Georgia, Augusta University, Augusta, GA, United States; ^2^Department of Neurology, Medical College of Georgia, Augusta University, Augusta, GA, United States; ^3^Department of Oral Biology and Diagnostic Sciences, Dental College of Georgia, Augusta University, Augusta, GA, United States

**Keywords:** intracerebral hemorrhage, hematoma, remote ischemic conditioning, neurological outcome, stroke, Rankin Scale

## Abstract

Spontaneous Intracerebral hemorrhage (ICH) is a devastating injury that accounts for 10–15% of all strokes. The rupture of cerebral blood vessels damaged by hypertension or cerebral amyloid angiopathy creates a space-occupying hematoma that contributes toward neurological deterioration and high patient morbidity and mortality. Numerous protocols have explored a role for surgical decompression of ICH *via* craniotomy, stereotactic guided endoscopy, and minimally invasive catheter/tube evacuation. Studies including, but not limited to, STICH, STICH-II, MISTIE, MISTIE-II, MISTIE-III, ENRICH, and ICES have all shown that, in certain limited patient populations, evacuation can be done safely and mortality can be decreased, but functional outcomes remain statistically no different compared to medical management alone. Only 10–15% of patients with ICH are surgical candidates based on clot location, medical comorbidities, and limitations regarding early surgical intervention. To date, no clearly effective treatment options are available to improve ICH outcomes, leaving medical and supportive management as the standard of care. We recently identified that remote ischemic conditioning (RIC), the non-invasive, repetitive inflation-deflation of a blood pressure cuff on a limb, non-invasively enhanced hematoma resolution and improved neurological outcomes *via* anti-inflammatory macrophage polarization in pre-clinical ICH models. Herein, we propose a pilot, placebo-controlled, open-label, randomized trial to test the hypothesis that RIC accelerates hematoma resorption and improves outcomes in ICH patients. Twenty ICH patients will be randomized to receive either mock conditioning or unilateral arm RIC (4 cycles × 5 min inflation/5 min deflation per cycle) beginning within 48 h of stroke onset and continuing twice daily for one week. All patients will receive standard medical care according to latest guidelines. The primary outcome will be the safety evaluation of unilateral RIC in ICH patients. Secondary outcomes will include hematoma volume/clot resorption rate and functional outcomes, as assessed by the modified Rankin Scale (mRS) at 1- and 3-months post-ICH. Additionally, blood will be collected for exploratory genomic analysis. This study will establish the feasibility and safety of RIC in acute ICH patients, providing a foundation for a larger, multi-center clinical trial.

## Introduction and Rationale

Intracerebral hemorrhage (ICH), the most common form of hemorrhagic stroke, accounts for up to 15% of all strokes, with 67,000 Americans suffering an ICH annually (Brown et al., 1996; Mayo et al., 1996; Broderick et al., 1999; Qureshi et al., 2001; Rincon and Mayer, 2004). One-year mortality rates are >60% and fewer than 20% of ICH patients recover functional independence after 6 months (Dennis et al., 1993; Gebel et al., 2002; Broderick et al., 2007). ICH is predominantly caused by the rupture of small vessels damaged by chronic hypertension or cerebral amyloid angiopathy. The subsequent extravasation of blood creates a space-occupying hematoma that induces local microvascular compression, brain tissue loss, and cerebrovascular dysfunction (Nehls et al., 1988; Qureshi et al., 2001; Fewel et al., 2003).

Hematoma volume is associated with neurological deterioration and high patient mortality, but no available treatment options significantly improve patient outcomes. The current American Heart Association Guidelines for the Management of Spontaneous ICH in Adults recommend surgical clot removal in neurologically deteriorating patients and in patients presenting with lobar clots > 30 mL and within 1 cm from the brain surface; however, the benefits of neurosurgical clot evacuation remain unclear for most ICH patients (Hemphill et al., 2015). Along these lines, the multi-center, randomized Surgical Trial in ICH (STICH) failed to observe an overall benefit of early surgical hematoma evacuation in supratentorial ICH, as compared to conservative management (Mendelow et al., 2005). Similarly, the effectiveness of stereotactic or endoscopic clot aspiration with thrombolytic usage remains uncertain (Hanley et al., 2019; de Havenon et al., 2020; Kellner et al., 2020). The lack of a clearly defined surgical benefit leaves medical management as the standard of care for most ICH patients, with the goal of stabilization until the clot intrinsically resolves. Unfortunately, this process may last several weeks/months, resulting in over 80% of survivors failing to recover long-term functional independence.

Transient bouts of sub-lethal ischemia, delivered either before or after an insult, increase tissue resiliency in multiple models of ischemia-reperfusion injury *via* a process deemed “ischemic conditioning” (Murry et al., 1986; Yellon and Hausenloy, 2005; Crisostomo et al., 2006). Remote-limb Ischemic Conditioning (RIC), the repetitive inflation-deflation of a blood pressure cuff on a limb, demonstrated safety and efficacy in clinical trials for myocardial infarction (Botker et al., 2010), intracranial stenosis (Meng et al., 2012), acute ischemic stroke (England et al., 2017), cerebral small vessel disease (Wang et al., 2017), and subarachnoid hemorrhage (Koch et al., 2011; Gonzalez et al., 2013, 2014). We first reported that RIC accelerated spontaneous hematoma resolution and improved long-term outcomes in two experimental models of ICH (Vaibhav et al., 2018). The beneficial effect of RIC was associated with activation of 5′-AMP activated protein kinase (AMPK), an immunometabolic switch that stimulated anti-inflammatory polarization of circulating myeloid cells, resulting in enhanced phagocytosis and an increased rate of clot resorption (Vaibhav et al., 2018). In addition to these acute benefits, chronic daily RIC attenuated cerebral white matter injury and improved neurological outcomes for months after ICH (Vaibhav et al., 2018).

Our pre-clinical data provided the conceptual framework for the open-label, assessor-blinded, randomized controlled Remote Ischemic Conditioning for Intracerebral Hemorrhage (RICH-1) trial (ClinicalTrials.gov, NCT03930940), which found a 7-day protocol of RIC was safe, well-tolerated, and accelerated hematoma resolution rate in 40 patients with supratentorial ICH (Zhao et al., 2020, 2021). Given these promising findings, the goal of this pilot study is to test the overarching hypothesis that unilateral RIC is a safe, feasible therapy to accelerate hematoma resorption and improve outcomes in ICH patients.

## Methods

### General Study Design

**FIGURE 1:**
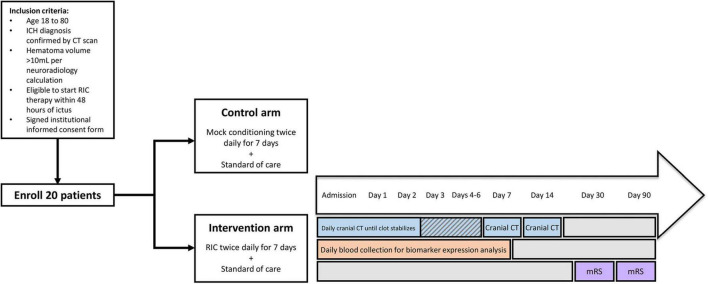
Study design. ICH patients meeting inclusion/exclusion criteria will be enrolled in the study after providing informed consent. Subjects will be randomly assigned to experimental arms [mock conditioning or unilateral daily remote ischemic conditioning (RIC) in addition to current standard of care]. Cranial computed tomography (CT) scans will be obtained on a daily basis until the clot stabilizes and then again at days 7 and 14. Blood will be collected daily in the first week for biomarker assessment. One month and 3 months post-ICH functional outcomes will be assessed by the modified Rankin Scale (mRS).

A pilot randomized, open-label, assessor-blinded controlled trial will be conducted at the Comprehensive Stroke Center at Augusta University Medical Center (Augusta, GA). All patients admitted to the neuro-intensive care unit and meeting inclusion/exclusion criteria will be eligible for enrollment. A total of twenty patients that meet pre-defined study criteria and providing informed consent will be randomized to either standard of care plus mock arm conditioning or standard of care plus arm RIC (4 cycles × 5 min inflation/5 min deflation per cycle) groups. Intervention will be initiated within 48 h of ICH onset and will be continued twice daily for seven consecutive days. The primary outcome measure will be safety. Secondary outcome measures will include hematoma volume and functional outcomes, as assessed by the modified Rankin Scale (mRS) at 1- and 3-months post-ICH. Cranial computed tomography (CT) scans will be obtained upon admission and daily thereafter until stabilization of clot expansion. CT scans will additionally be obtained at 7- and 14-days post-admission to monitor hematoma volume. Arterial blood will be collected over the first 7 days post-ICH and used for exploratory biomarker analysis. The study design, as depicted in Figure 1, was approved by the Augusta University Institutional Review Board (IRB-ID: 1525689-2).

### Patient Population: Inclusion and Exclusion Criteria

Computed tomography scans will be reviewed with the patient, when possible, or the person authorized to sign on behalf of the patient based on institutional criteria.

#### Inclusion Criteria

• Age 18–80 of both genders.
• Diagnosis of ICH confirmed by CT scan.
• Hematoma volume > 10 mL per neuroradiology calculation.
• Eligible to start RIC therapy within 48 h of ictus (i.e., onset of symptoms).
• Signed informed consent.

#### Exclusion Criteria

• Intracerebral hemorrhage associated with trauma, tumor, ruptured aneurysm, or arteriovenous malformation, venous infarct/venous sinus thrombosis, or post-procedural (e.g., post-intraarterial tissue plasminogen activator).
• Planned surgical intervention within 48 h of ictus.
• Impending herniation (shift > 10 mm) or clinical signs of brainstem dysfunction.
• Severe hepatic or renal dysfunction.
• Life expectancy is <3 months secondary to other comorbid conditions.
• Inability to administer RIC for any reason.

### Randomization

Enrolled patients will be randomized in a 1:1 ratio into two study arms (mock conditioning control group or RIC group). The randomization sequence will be generated by a computer program and the group assignment will be coded by a research coordinator. Assessments of CT scans, neurological outcomes, and biomarker analysis will be performed by qualified investigators blinded to treatment assignments. At study conclusion, patient data will be decoded and analyzed.

### Interventions

Routine standard medical care will be provided to all subjects, in accordance with the American Heart Association Guidelines for the Management of Spontaneous ICH (Hemphill et al., 2015). Briefly, the goal is to prevent hemorrhage progression, reduce intracranial pressure, and support the patient medically while the brain is recovering. Tracheal intubation is performed for patients that are unable to protect the airway (rapidly deteriorating mental status or GCS ≤ 8). Oxygenation, blood pressure control, CSF diversion, and intracranial pressure management are performed, as required. Additionally, anticoagulant or antiplatelet medications are immediately discontinued and medication specific reverse anticoagulation is administered. We also provide deep vein thrombosis (DVT) prophylaxis with intermittent pneumatic compression initially. After 24 h of stable scans, we initiate low-molecular weight heparin (LMWH) and if the patients have a particular medical risk factor (e.g., artificial valve, DVT) anti-coagulation is restarted.

**FIGURE 2:**
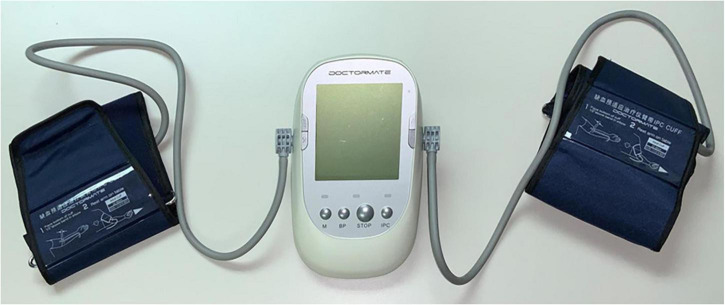
Remote ischemic conditioning (RIC) device. The RIC device is comprised of blood pressure cuffs attached to an automated controller that is programmed to deliver four cycles of 5-min inflation/5-min deflation. The RIC intervention group receives inflation to a cuff pressure of 200 mm Hg. The mock conditioned group receives cuff placement, but without inflation. The cuff is placed on the upper arm by trained medical staff.

The study will incorporate two study arms. The **Control arm** will receive current standard of care plus unilateral, twice-daily mock conditioning whereby a cuff will be placed on an upper limb, but inflation/deflation will not be performed. The **Intervention arm** will receive current standard of care plus unilateral, twice daily RIC. RIC will be performed by placing an automated blood pressure cuff on the upper arm (Figure 2). The cuffs will deliver four cycles of 5 min compression (cuff-inflated) with a cuff pressure of 200-mm Hg followed by 5 min of reperfusion with the cuff deflated. The total treatment time will be 35 min. This procedure will be performed twice daily (once in the morning, one in the evening) and will be continued for 7 days from the time of admission.

### Outcomes

#### Safety Outcomes Assessment

Remote ischemic conditioning is a non-invasive therapy with documented safety in critically ill neurological injury patients (Koch et al., 2011; Gonzalez et al., 2013, 2014; Mayor et al., 2013; England et al., 2017). The device consists of a standard blood pressure cuff controlled by an automated device that regulates both cuff pressure and timing of inflation/deflation. Throughout the trial, patients will be monitored for the development of unexpected adverse events during the study period.

#### Efficacy Outcomes Assessment

The primary study outcome is hematoma volume, as assessed by cranial CT scanning. Volumetric data from CT scans will be obtained at the time of admission and daily thereafter until clot expansion has stabilized on two successive CT scans. We will then obtain ratios of clot volume to map the rate of clot progression [i.e., clot volume (V_*c*_) day 3/V_*c*_ day 2, V_*c*_ day 2/V_*c*_ day 1]. Volumetric data obtained at 7- and 14-days post-admission will be divided by the maximum clot volume (around day 3). Images will be quantified by neuroradiologists blinded to group randomization. Secondary study outcome is functional outcome, as assessed by modified Rankin Scale (mRS) score at 1- and 3-months post-ICH. The mRS is a well-characterized measure of functional status. Patients will be grouped based on good functional outcomes (mRS 0–2) or poor functional outcomes (mRS 3–6). Neurological assessments will be conducted by physicians that are blinded to group randomization.

Exploratory studies will use flow cytometry and genomic profiling of patient blood. Discarded whole blood samples will be obtained from the blood bank following routine draws as a part of standard care. Metabolic and inflammatory markers will be assessed in myeloid cells, paralleling our pre-clinical studies that showed RIC increased anti-inflammatory macrophage polarization to stimulate phagocytosis of extravasated erythrocytes (Vaibhav et al., 2018). Remaining samples will be used for discovery-based pharmacodynamic biomarker identification to monitor the biological responses to RIC and to establish a framework for future mechanistic studies.

#### Data Monitoring

All coded study data will be electronically uploaded to an encrypted server. An investigator blinded to group assignment will manage the data and perform statistical analyses.

#### Statistical Analyses

This is a proof-of-concept pilot study and the number of patients will not achieve statistical power. Data comparisons will be made using GraphPad Prism Software. Categorical variables will be analyzed using the chi-squared test and the continuous variables will be assessed using the independent *t*-test. A *p*-value of <0.05 will be considered significant.

## Discussion

Intracerebral hemorrhage is devastating neurological injury that produces high patient mortality and significant loss of quality of life in survivors. The rupture of cerebral small vessels damaged by hypertension or cerebral amyloid angiopathy creates a dynamically expanding, space-occupying intracerebral hematoma that disrupts neurological function. The persistence of the clot within the brain parenchyma for days and weeks further induces iron-mediated oxidative stress, exacerbating cerebrovascular function and inducing permanent brain tissue loss.

Hematoma volume reduction to reduce local and regional mass effect, improve cerebral perfusion, and accelerate clot resorption remains a long-standing goal of ICH management; however, the clinical benefits of surgical clot removal remain unclear. The international, multi-center STICH trial concluded the benefits of early surgical intervention were not statistically different from intensive medical management alone in the majority of ICH patients, suggesting a limited benefit of surgery after ICH. More recent studies focused on minimally invasive surgery plus infusion of recombinant tissue plasminogen activator (rtPA) approaches show promise in early-stage clinical trials; however, the Minimally Invasive Surgery Plus Alteplase for Intracerebral Hemorrhage Evacuation III (MISTIE-III) trial observed a reduction in hematoma volume without significant functional improvement (Hanley et al., 2019). This lack of a clear benefit from surgical interventions to reduce hematoma volume coupled with a dearth of medical/pharmacological treatment options, leaves supportive care as the key treatment approach for ICH patients.

We identified that RIC accelerated hematoma resolution, reduced peri-hematoma edema, improved cerebral blood flow, limited white matter loss, and improved neurological outcomes following ICH in rodents (Vaibhav et al., 2018). Interestingly, the beneficial effects of RIC were dependent, at least in part, on circulating factors, including anti-inflammatory polarization of myeloid cells that accelerated CD36-mediated phagocytosis of extravasated erythrocytes within the brain parenchyma (Vaibhav et al., 2018). Thus, RIC may improve ICH outcomes, including hematoma resolution, *via* pleiotropic mechanisms that could prove more efficacious than surgical evacuation. Moreover, RIC could provide a potential adjunct therapy in conjunction with minimally invasive surgery and tPA.

A potential strength of this study is the high number of ICH patients seen in our institution. Our medical center is located in the South Eastern United States, a region of the country dubbed the “stroke belt,” due to an elevated incidence of stroke including ischemic stroke, subarachnoid hemorrhage, and spontaneous ICH. Our institution treats an average of 840 stroke diagnoses per year with 120 (14%) of these patients having an intracerebral hemorrhage. Due to our regional demographics, we see a larger percentage of younger and African-American patients, which will allow our study to address ICH treatment in these less studied populations. A limitation of our study is that the proposed protocol is based on pre-clinical work; however, it remains unknown whether this proposed protocol is optimal for humans, as compared to rodents. To partially address this point, we will monitor changes in macrophage activation to determine whether this RIC effect parallels that observed in rodents. To avoid bias with respect to data interpretation, scans and neurological scale rating will be performed by investigators blinded to experimental groups; however, given the nature of RIC, subjects will be aware of their experimental group. Nonetheless, our pilot data will provide feasibility and safety of RIC in ICH patients.

## Conclusion

The proposed clinical pilot study will provide early-stage safety and feasibility data to indicate whether RIC may non-invasively accelerate spontaneous hematoma resolution and improve ICH patient outcomes. This knowledge will establish a crucial foundation for larger, multi-center clinical studies of efficacy.

## Data Availability Statement

The original contributions presented in the study are included in the article/supplementary material, further inquiries can be directed to the corresponding author.

## Ethics Statement

The studies involving human participants were reviewed and approved by Augusta University Institutional Review Board (IRB). The patients/participants provided their written informed consent to participate in this study.

## Author Contributions

AJ, KD, and JV conceptualized the study and drafted the manuscript. All authors contributed to experimental design and reviewed the final version of the manuscript.

## Conflict of Interest

The authors declare that the research was conducted in the absence of any commercial or financial relationships that could be construed as a potential conflict of interest.

## Publisher’s Note

All claims expressed in this article are solely those of the authors and do not necessarily represent those of their affiliated organizations, or those of the publisher, the editors and the reviewers. Any product that may be evaluated in this article, or claim that may be made by its manufacturer, is not guaranteed or endorsed by the publisher.
